# Rewarding behavior with a sweet food strengthens its valuation

**DOI:** 10.1371/journal.pone.0242461

**Published:** 2021-04-14

**Authors:** Jan M. Bauer, Marina Schröder, Martina Vecchi, Tina Bake, Suzanne L. Dickson, Michèle Belot

**Affiliations:** 1 Department of Management, Society and Communication, Copenhagen Business School, Frederiksberg, Denmark; 2 Faculty of Economics and Management, Leibniz University Hannover, Hannover, Germany; 3 Department of Agricultural Economics, Sociology, & Education (AESE), The Pennsylvania State University, University Park, Pennsylvania, United States of America; 4 Department of Physiology/Endocrine, Institute of Neuroscience and Physiology, The Sahlgrenska Academy at the University of Gothenburg, Gothenburg, Sweden; 5 Department of Economics, Cornell University, Ithaca, New York, United States of America; Tokai University, JAPAN

## Abstract

Sweet foods are commonly used as rewards for desirable behavior, specifically among children. This study examines whether such practice may contribute to reinforce the valuation of these foods. Two experiments were conducted, one with children, the other with rats. The first study, conducted with first graders (*n = 214*), shows that children who receive a food reward for performing a cognitive task subsequently value the food more compared to a control group who received the same food without performing any task. The second study, conducted on rats (*n = 64*), shows that rewarding with food also translates into higher calorie intake over a 24-hour period. These results suggest that the common practice of rewarding children with calorie-dense sweet foods is a plausible contributing factor to obesity and might therefore be ill advised.

## Introduction

Poor nutrition is associated with three of the five highest risks for mortality in the world: high blood pressure; high blood glucose; and obesity [1]. Dietary habits are known to form in childhood [2, 3], and are strongly influenced by individual taste valuations [4, 5]. Identifying key factors contributing to the formation of tastes for foods that are high in fat and sugar is therefore paramount to address the ongoing obesity crisis. In this study, we examine the role of using food as a reward in the formation of tastes.

In a representative survey among families from five countries, we find that food is commonly used to reward behavior (see Supporting information). However, there is little and only correlational evidence on how such practices affect the valuation of the food used as a reward [6, 7]. We argue that using food as reward might reinforce its valuation for two possible reasons. First, the theory of evaluative conditioning proposes that the value of a conditioned stimulus is affected by previous pairing with another positive or negative unconditioned stimulus [8, 9]. While conditioning is proposed as a central driver that shapes food preferences [10], there is only limited empirical support for this link in humans [11–13]. Second, studies conducted in non-food related contexts have demonstrated that the valuation of an object increases with the effort provided to obtain this object [14]. The common explanation for the increase in valuation is the psychological mechanism of *cognitive dissonance* [15], which is the notion of psychological discomfort that may arise in the presence of inconsistent beliefs or value. In a context where effort has to be provided, it is discomforting if the effort does not lead to a greater reward. Increasing the valuation of an object used as a reward may therefore occur to resolve cognitive dissonance. Such effect of effort justification has already been documented in children regarding the valuation of objects other than food [16]. A similar phenomenon might occur with food valuations when effortful behavior is rewarded with this food.

We present evidence from two experiments. In a first experimental study, with children aged six to eight, we find evidence that using a sweet food (a piece of dried apple) as a reward over a period of four consecutive weeks increases the valuation of this food. In a second experimental study, with rats, we show that using a sweet food as a reward increases the consumption of this food and in addition, we show that the increased consumption of the food used as a reward is not compensated by a decrease in consumption of other foods. Rather, we observe a significantly higher calorie intake over a period of 24 hours. This latter result is difficult to demonstrate in humans because of monitoring and measurement issues, and justifies the combination of studies. Our study adds to previous research on mechanisms effecting food valuation in animal studies [17, 18]. To the best of our knowledge, no study using a related paradigm has focused on the 24-hour calorie intake.

Based on these results, we conclude that the practice of using sweet and calorie-dense foods as rewards can be a contributing factor to obesity and should therefore be discouraged. Further research is needed to demonstrate whether pairing healthier foods (such as fruit and vegetables) with rewards could increase their appeal and could contribute to the formation of healthier food preferences.

## Materials and methods

### Experiment 1 (children)

The study was reviewed and approved by the Ethics Committee of the University of Edinburgh. We conducted the first study with a sample of 214 school children aged six to eight, enrolled across three primary schools in Cologne. In each school, classes were randomly assigned to one of two treatment or the control group. Eleven classes took part in total. We conducted nine visits per class in the period between April and July 2016. During the first four weeks, we visited the schools twice per week, with a follow-up visit after one month. The study protocol included three assessment sessions (visit 1, 8 & 9) and six treatment sessions (visits 2 to 7).

During the treatment sessions, all children received a piece of dried apple on two occasions during each week. As it is well established that mere exposure to a food strengthens its valuation [12], a control group of children received the food “for free”, while children in the treatment groups received the food as a reward for completing a counting task. All groups were visited at the same frequency. In the control group classes, children received the reward food, a dried piece of apple, unconditionally ("Here, have a piece of apple"). In the treatment classes, children were asked at each visit to complete a cognitive task and received the dried apple as a reward for completing the task. The task consisted of counting a random number of dots in several different pictures. The number of pictures varied between the two treatment groups: in the low effort group children had to count dots in two pictures, while in the high effort group they were required to count dots in ten pictures. Varying the number of pictures allows us to identify the role of effort in driving a change in valuation for dried apple. We ensured a balanced exposure to the reward item between the groups by providing assistance to those who had difficulties with the effort task. Consequently, all children received a reward from the experimenter, saying: "Well done, here is your reward!".

At three points in time, we assessed the valuation of the dried apple: before (*baseline*), immediately after (*short-term*) and four weeks after the interventions (*long-term*). We returned to the schools four weeks after the intervention to evaluate whether the changes in valuation persisted over time. The valuation of the dried apple is assessed with three different measures: First, children sampled the dried apple and three other snacks (a dried apricot, a cracker & a natural yogurt) and ranked their liking on a four-point scale ranging from a sad to a happy emoticon (*liking*) (examples are shown in the Supporting information). Second, children were asked to conduct five comparisons of liking between two of the snacks, including two additional hypothetical comparisons to French fries and gummy bears (*comparison*). Third, children were asked to choose their overall preferred snack from four options (the dried apple and three alternatives). This choice was incentivized as children received their chosen snack at the end of the assessment session (*choice*). We chose the four snacks based on pilot sessions in two classes (not involved in the experiment). After assessing children’s valuation for ten snacks using the three outcome measures used in the experiment (dried apples, dried apricots, dried prunes, wholegrain crackers, rice cakes, carrot sticks, pears, grapes, drinking yoghurt and natural yogurt), we chose the four snacks with similar and more moderate rating.

The data was analyzed with STATA 16. The descriptive statistics in S1 Table show no differences between the groups in terms of child characteristics (age and sex) or valuation of the food reward in all three measures at baseline, suggesting that despite a small number of classes the randomization resulted in balanced groups. Results from S1 Table also show that refusal of trying the reward and attendance do not vary between treatments and unlikely confound our results. Neither exposure nor participation in the treatment groups differs from the control. To keep the sample consistent between all analytical steps, we only include children present during all three assessments. Such a complete case analysis yields unbiased estimates as long as observations are missing at random [19]. In support of this assumption, we fail to predict missing values based on group assignment or preference at baseline (see S2 Table).

Our main strategy of analysis relies on regression models to account for the panel and nested structure of our data. Considering each class as a cluster, we find the intra cluster correlation (ICC) within the classes to be generally low: for all outcome measures the ICC never reaches a value above 0.1 (baseline ICC: valuation<0.001; choice = 0.02; comparison = 0.09). However, a viable method using a bootstrapping technique to account for the comparably small number of clusters is unavailable for ordered response models. We therefore use ordinary least squares (OLS) [20]. Compared to the regular cluster method [21], the use of wild bootstrap clusters slightly increases the standard errors and results into the loss of statistical significance for some measures. We provide alternative methods to estimate the treatment effect and the standard errors in the Supporting information.

### Experiment 2 (rats)

We conducted the second study with 64 male *Sprague-Dawley rats* (Charles River, Sulzfeld, Germany). All rats were young adults (seven weeks of age at arrival). Prior to experimental procedures, the rats were allowed to acclimatize in group-housing for one week and in single housing for another week in which they were handled daily to avoid stress. The rats were kept under standardized non-barrier conditions on a 12:12 hour light-dark-cycle (with lights on from 7.00 to 19.00) at a temperature of approximately 21°C and a humidity of approximately 50%. They had ad libitum access to standard maintenance chow (\#801151 RM1-P; Special Diets Services, Witham, UK; 17.5% protein, 75.0% carbohydrate, 7.5% fat by energy, 2.57 kcal/g) and water unless otherwise stated. All animal experimental procedures were approved by the local animal ethics committee at the University of Gothenburg.

After a further week in single-housing in which baseline measures were taken, rats were divided into two groups according to body weight (333.9 ± 2.5 g), 24 hour chow intake (26.2 ± 0.4 g) and three hour sucrose intake (2.7 ± 0.2 g; #1811251, 45 mg sucrose pellets; TestDiet, Richmond, IN, USA; 3.4 kcal/g). Rats were then either conditioned to press a lever for sucrose pellets in operant conditioning chambers (Med-Associates Inc.; St Albans, Vermont, USA) using a progressive ratio (PR) paradigm (treatment rats, n = 32), or they were placed in the chambers but received the same number of sucrose pellets for free calculated from the average number of pellets earned by the treatment rats the previous day (control rats, n = 32). Each chamber had a metal grid floor, a house light, two retractable levers with cue lights above them, and a pellet dispenser that can deliver 45 mg pellets. Only one of the levers was active during conditioning for the treatment rats; pressing the inactive lever had no programmed consequence. The chambers for the control rats were programmed so that only the house light would be on during the session, levers were retracted and cue lights were off. Operant conditioning under PR is a commonly used method to induce motivation for food such as sucrose pellets [22, 23]. The conditioning was done in two stages: fixed ratio (FR) and progressive ratio (PR). The conditioning in the FR stage was done under mild food restriction (with access to 15 g of chow per day at the beginning of the dark phase) with a schedule of 30 min FR1 (over 3 days), FR3 (over 2 days), and FR5 (over 2 days) sessions twice a day (one session in the early light phase and the other session in the mid to late light phase). In FR1, rats learn to press the lever once to get a pellet, in FR3 three times for one pellet and in FR5 five times for one pellet. When all rats managed to earn at least 50 pellets in one FR level, they progressed to the next FR level during the next session. During FR sessions the operant chambers were programmed to shut down automatically if a rat earned the maximum amount of a 100 pellets.

Once the rats had passed the FR stage, they progressed to the PR stage. This consisted of 90 min PR sessions with mild food restriction once a day over 3 days, followed by 90 min PR sessions without food restriction once a day over 8 days. Performance on the PR schedule was considered stable when the number of rewards (sucrose pellets) earned in a 90 min session deviated by ≤10% for at least 3 consecutive days. In each PR session the number of presses required to receive the next reward was progressively increased in order to determine the amount of work the rat is willing to put into obtaining the reward. The response requirement increased according to the following equation: response ratio = [5e ^(R*0.2)^]– 5 whereby R is equal to the number of rewards already earned plus 1 (the next reward). It went through the following series: 1, 2, 4, 9, 12, 15, 20, 25, 32, 40, 50, 62, 77, 95, 118, 145, 178, 219 and so on. The final completed response ratio is the breakpoint, i.e. when a rat ceased to engage in lever-pressing and fails to earn any more rewards within 60 min. During PR sessions the operant chambers were therefore programmed to shut down automatically if a rat had failed to earn a reward within 60 min, but all rats remained in the operant chambers for the entire session length of 90 min regardless of the session finishing earlier. On average the sessions were ending after 73.9 ± 0.7 minutes. During all PR sessions we measured the active/inactive lever presses, the response ratio (breakpoint) and the number of sucrose pellets earned. During the last 3 days of PR conditioning, rats pressed the active lever 123.8 ± 11.1 times, with a response ratio of 35.8 ± 2.6 and received 8.0 ± 0.3 sucrose pellets. The number of sucrose pellets earned by the treatment rats during the last stage varied between 4 and 12, the control rats received the average amount of pellets for free. Chow and water were withheld during all conditioning sessions.

Two days after the last PR training session, we investigated whether the amount of sucrose pellets eaten was dependent on whether the rats had to previously push a lever to receive sucrose (treatment group) or not. We placed all rats into the conditioning chambers for three hours with freely available sucrose pellets. Moreover, we also measured chow intake during the rest of the 24-hour day to see whether increasing the value of the sucrose by the reward paradigm affected the intake of chow and overall calorie intake. We compare overall intake between the groups when all rats were offered sucrose pellets ad libitum over 3 hours. For one rodent in the reward group no sugar consumption data was available. Data were analyzed in Excel and STATA (version 16) primarily using independent samples T-tests.

## Results

### Experiment 1 (children)

**Fig 1:**
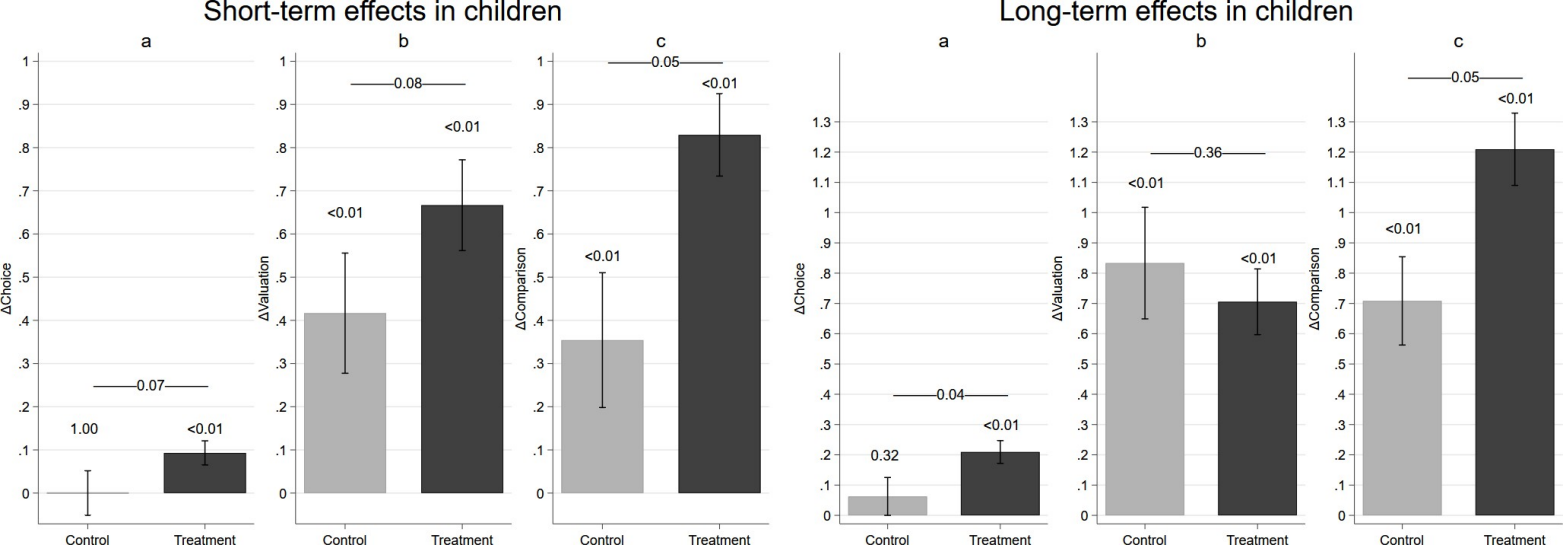
Short- and long-term effects of the treatment. Left panel displays the estimates for the second assessment (short-term effect) for treated and untreated children. Right panel displays the estimates for the third assessment (long-term effects) for treated and untreated children. The bars represent the linear predictions from OLS for the three valuation measures.–a. choice: proportion of children choosing the dried apple, b. liking: liking of the dried apple measured on a 4-point scale, and c. comparison: number of times the dried apple is preferred in 5 pairwise comparisons. P-values of group differences are reported above the bars. Standard errors are clustered on the class level. The full models are presented in S3 Table. Data are presented as mean/proportion ± 95% CIs. While the concept of evaluative conditioning would not make a clear prediction about the differences between the two treatment groups, the theory of effort justification predicts that children who must provide high effort should increase their valuation more, relative to those who only have to provide low effort to receive the food reward. To investigate whether this is the case, we compare the increase in valuation between the two effort treatments by estimating linear regression models including a variable for ‘high effort treatment’. This coefficient corresponding to this variable should capture any differential effect between the two treatment groups. We find a small and not statistically significant estimated coefficient in most regressions. For the c*omparison* measure, contrary to the predictions of effort justification, we find a negative differential effect in the short-term (*n*_*total*_ = 177, main effect: *B* = 0.57, SE_clustered_ = 0.12, *t* = 4.57, *P*<0.01; high effort: *B* = -0.25, SE_clustered_ = 0.12, *t* = -2.07, *P* = 0.07; see S7 Table).

We estimate the impact of receiving food as a reward by estimating linear regression models for the three food valuation measures in the second and third assessment separately. The main results are presented in Fig 1. The left panel displays the mean predictions from the linear regression model for the second assessment (short-term effects). The mean predictions are presented separately for the control group (light grey) and the treatment groups (black). For all three measures, we find that children who had to work to receive the food valued it on average more than the control group, who received the apple unconditionally [n_total_ = 177; OLS; *choice*: *B* = 0.08, *SE* = 0.03, *t* = 2.98, *P* = 0.01; *liking*: *B* = 0.28, *SE* = 0.10, *t* = 2.76, *P* = 0.02; *comparison*: *B* = 0.45, *SE* = 0.13, *t* = 3.53, *P* = 0.01; see S3 Table]. These findings are supported by the binary and ordered response models [n_total_ = 177; *choice*: Logit, odds ratio (*OR)* = 2.49, *SE* = 0.59, *z* = 3.83, *P*<0.01; *liking*: Ologit, *OR* = 2.44, *SE* = 0.56, *z* = 3.86, *P*<0.01; *comparison*: Ologit, *OR* = 2.06, *SE* = 0.58, *z* = 2.59, *P* = 0.01, see S4 Table].

The right panel of Fig 1 displays the mean predictions from the linear regression model for the third assessment (long-term effects). We observe a long-term treatment effect for *choice* [OLS, *n*_*total*_ = 177, *B* = 0.14, *SE* = 0.04, *t =* 63.84, *P*<0.01] and *comparison* [OLS, *n*_*total*_ = 177, *B* = 0.47, *SE* = 0.09, *t* = 5.34, *P*<0.01], but no significant difference between the groups for the measure of *liking* [OLS, *n*_*total*_ = 177, *B* = -0.15, *SE* = 0.20, *t* = -0.78, *P* = 0.46; see S3 Table]. Similarly to the short-term effects these findings were replicated with non-linear response models [n_total_ = 177; *choice*: Logit, *OR* = 2.49, *SE* = 0.46, *z* = 4.93, *P*<0.01; *liking*: Ologit, *OR* = 0.69, *SE* = 0.33, *z* = -0,76, *P* = 0.45; *comparison*: Ologit, *OR* = 1.71, *SE* = 0.30 *z* = 3.01, *P*<0.01, see S4 Table]. Non-parametric estimates comparing the increase in valuation between both groups provide comparable results (see S5 Table).

Additionally we find evidence for an exposure effect. The mere fact that children repeatedly consume a food already increases the valuation of the food. Comparing the three assessments for children in the control group, we observe a significant increase for two of the three measures between the first assessment (baseline) and the second assessment (short-term) [n_total_ = 48; *choice*: Logit, *OR* = 1.00, *SE* = 0.68, *z* = 0.00, *P* = 1.0; *liking*: Ologit, *OR* = 1.7, *SE* = 0.35, *z* = -2.60, *P*<0.01; *comparison*: Ologit, *OR* = 2.30, *SE* = 0.67 *z* = 2.84, *P*<0.01], as well as between the first and third assessment [n_control_ = 48; *choice*: Logit, *OR* = 1.89, *SE* = 1.21, *z* = 0.99, *P* = 0.32; *liking*: Ologit, *OR* = 3.46, *SE* = 1.03, *z* = 4.18, *P*<0.01; *comparison*: Ologit, *OR* = 4.99, *SE* = 1.63 *z* = 4.90, *P*<0.01; see S6 Table]. These results are also supported by non-parametic estimations, see S5 Table.

### Experiment 2 (rats)

**Fig 2:**
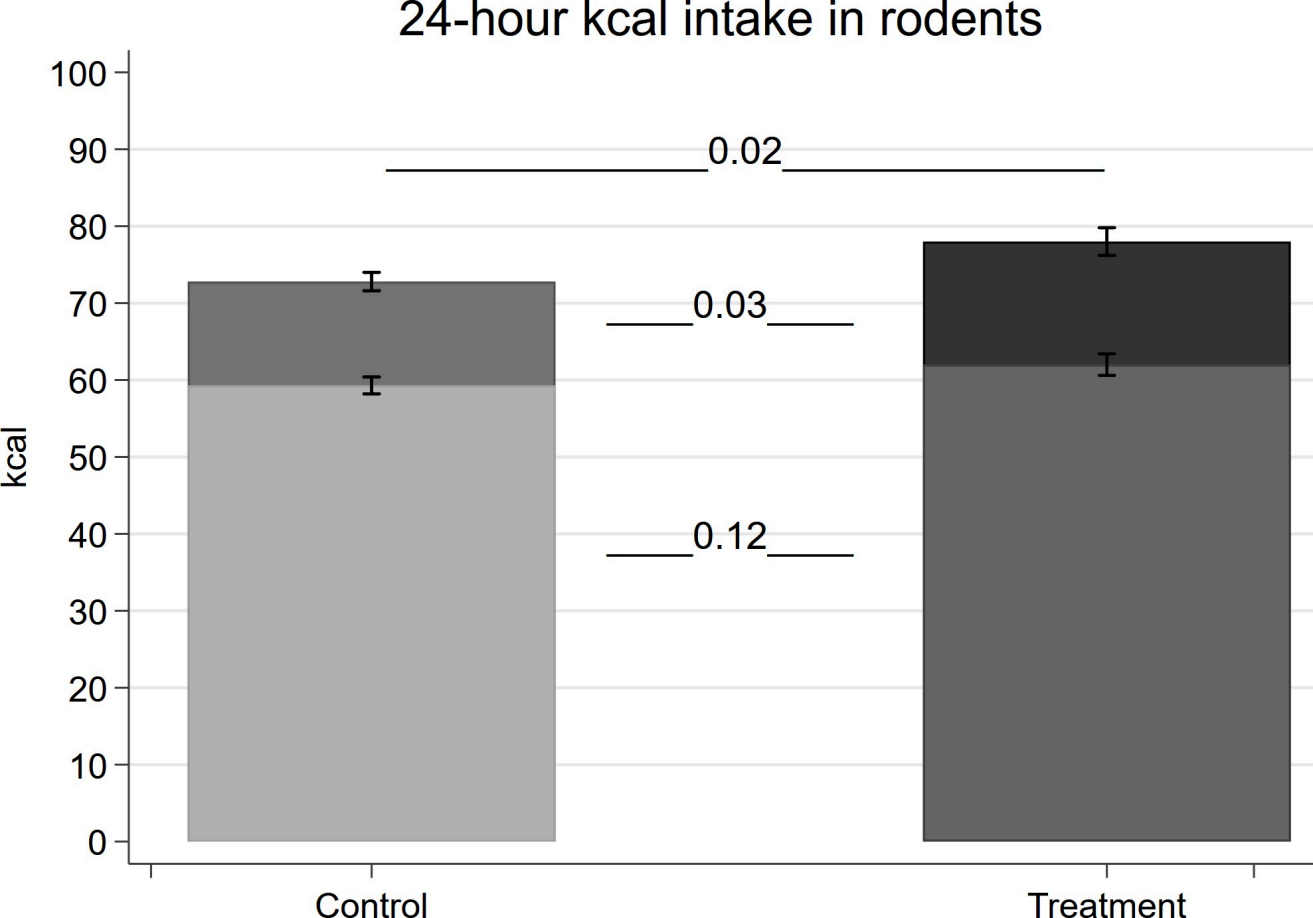
Consumption measures of rats. Energy intake (kcal) of rats, lighter areas in both bars indicate chow intake, darker areas above indicate sucrose intake from the first 3 hours over. Results from independent sample t-tests are presented. P-values between both foods are placed between the bars. The p-value for the overall kcal difference in 24 hours is placed above the bars. Data are presented as mean ± 1 SEM.

We find that rats in the treatment group eat on average 0.87g (2.97 kcal) more sucrose pellets over three hours than the control rats [T-Test, *n*_*control*_ = 32, *n*_*treatment*_ = 31, *t* = 2.21, *P* = 0.03; Fig 2]. We do not find evidence that rats in the treatment group compensate for the increased sucrose intake by consuming less chow. Instead, we observe even a slightly larger chow intake of rats in the treatment group compared to those in the control group [T-Test, diff = 2.75 kcal, *n*_*control*_ = 32, *n*_*treatment*_ = 32, *t* = 1.58, *P* = 0.12]. Overall, we find that the 24-hour calorie intake of the treatment group is significantly larger than that of the control group [T-Test, diff = 5.2 kcal, *n*_*control*_ = 32, *n*_*treatment*_ = 32, *t* = 2.36, *P* = 0.02] (see Fig 2).

For the rats in the treatment group, we observed a positive relationship between measures from the PR sessions (mean of the last 3 sessions) and the *ad libitum* sucrose intake over the 3 hours. The higher the sucrose intake, the more effort was excerted during the conditioning process measured by: the number of active lever presses (Pearson correlation, *n*_*treatment*_ = 31, *P* = 0.010), the response ratio (Pearson correlation, *n*_*treatment*_ = 31, *P* = 0.011) and the number of sucrose pellets earned (Pearson correlation, *n*_*treatment*_ = 31, *P* = 0.029).

While the PR sessions did not lead to an overall difference in the body weight of rats between the two groups measured regularly during the conditioning process (repeated measures ANOVA, conditioning as the between-subject factor: P = 0.927), the average weight of the rats in the treatment group was slightly higher than those in the control group measured directly before their *ad libitum* access to sucrose [T-Test, diff = 8.9g, *n*_*control*_ = 32, *n*_*treatment*_ = 32, *t* = 1.78, *P* = 0.08]. Controlling for the difference in body weight reduces the treatment effect for group differences in sucrose intake by one fourth, with a p-value of 0.08 instead of 0.03. Similarly, differences in 24-hour calorie intake reduced from 5.2 kcal [OLS: *n*_*total*_ = 64, t = 2.36, P = 0.02] to 3.46 kcal [OLS: *n*_*total*_ = 64, t = 1.76, P = 0.08].

## Discussion

In two experiments, we show that using a sweet food as a reward increases its subsequent appeal. The results from the experiment with children are consistent with the theory of *evaluative conditioning* whereby the value of a conditioned stimulus (here a food used as a reward) is affected by previous pairing with another positive or negative unconditioned stimulus [8, 9]. While conditioning is proposed as a central driver in the formation of food preferences [10], our study is the first to provide evidence that rewarding with food reinforces its valuation.

An important question is whether the effect of using food as a reward on valuation translates into an increase in overall calorie intake. Children may be more inclined to consume the food used as a reward but, triggered by homeostatic regulators, their consumption of other foods might be reduced such that the overall calorie intake remains unchanged [14]. In humans, it is challenging to obtain accurate measures of calorie intake over a longer period. Self-reported intake is usually imprecise and biased downwards, while direct monitoring is obtrusive and may distort normal eating behavior [24].

Our experimental study with rats allows us to evaluate whether using food as a reward affects the subsequent consumption of this food and the 24-hour calorie intake. By measuring chow intake post treatment and for a period of 24 hours, we evaluate whether increasing the value of the sucrose by the reward paradigm affects the intake of other foods. The results indicate that the increase in valuation of a reward leads to an increase in total calorie intake.

Combining the results of these two studies, we conclude that the common practice of rewarding children with sweet foods may be contributing factor to weight gain among children.

Matching an animal study with a human study is a challenging task. The experiments were both designed to test the hypothesis that rewarding with a (sweet) food changes the valuation of that food. The protocols differ, however, and we remain cautious in the translational conclusions that are possible to draw. Here we discuss three important differences between the two studies and briefly explain the choices made: 1) the human experiment was performed in children of both sexes, whereas the rat experiment was undertaken in adult males; 2) the schedules of reinforcement were different and 3), the food reinforcer was different.

In the rodent study, it was not an option to match the age and gender to that of the study in children. Rats are considered infants from birth until 3 weeks of age at which point they are weaned. The “juvenile period” occurs from 3 weeks to 8 weeks old and our experiment (that takes about 7 weeks including acclimatization, baseline, conditioning and testing phases) is not possible to undertake in this short time frame. Gender matching would only be relevant if we had the opportunity to explore the juvenile period in rats after which we would need to take into account the stage of the oestrus cycle.

Regarding the schedule of reinforcement, it should be noted that children can be explicitly told that their effort will be rewarded. The results from comparing the two treatment groups with different effort levels suggest that evaluative conditioning is a more plausible explanation for the observed treatment effect in children. Rats, however, need to learn the reinforcing value of a food by undertaking a motivational task (here, by pressing a lever). Given that effort was not manipulated in the animal experimental design, we cannot draw direct conclusions about the mechanisms for increased valuation for the rats.

Finally, regarding the choice of food reinforcer: for children, we chose a sweet palatable food. Dried apples comprise 56% sugar and have an energy density of 2.8 kcal/g. Rats are usually fed a grain-based diet. Foods rich in fat and/or sugar are considered as palatable or rewarding to rats. There are only a few options of food reinforcers available in tablet form, which are mostly either grain-based or sucrose-based. Therefore, our choice of reinforcer for the rats was sucrose-based.

The fact that both studies point to a similar effect, despite differences in protocols, is in our view evidence that the effect is likely to be robust. A question for further research is whether this mechanism of food preference formation extends to other tastes, non- sweet tastes in particular, and to foods that have a lower calorie content or a higher nutrient density. If that is the case, such mechanism may be exploited to design interventions aimed at fostering the formation of “healthier” food preferences, e.g. preferences for foods with a high nutrient calorie ratio.

## Supporting information

S1 File (DOCX)

S1 Fig Use of food reward. Share of participants reporting that in their family sweat treats are at least rarely used to reward for (1) doing something difficult, (2) doing something good or performing well, or (3) an achievement. Data are presented as mean ± 1 SEM.
(TIF)

S1 Table Summary statistics and initial levels. ^a^ Values based on a parental survey response with varying sample size (N): Control (35); Low-Effort (48); High-effort (47). ^b^ Average frequency of attendance to the 6 treatment sessions. ^c^ Average frequency of trying the food reward to the 6 treatment sessions. ^d^ Average value at baseline. ^e^ P-values based on a two-sample Wilcoxon rank-sum (Mann-Whitney) test for ordinal variables. Fisher’s exact test is used for binary variables.
(DOCX)

S2 Table Attrition. Notes: Linear probability model predicting missing values in the data. P-values below 0.1 in bold based on clustered on the class level in parenthesis. Binary dependent variable = 1 if at least 1 of 3 outcomes is missing during at least one assessment.
(DOCX)

S3 Table Main outcome. Notes: Dependent variables are: choice (change in percentage of children choosing the dried apple), liking (change in liking measured on a 4-point scale), and comparison (change in number of times the dried apple is preferred in 5 pairwise comparisons). Independent variables are the binary Treatment-group indicator and baseline values are the respective food valuations in the first assessment; we also control for school specific fixed-effects. Model based on OLS. P-values below the coefficients based on: () clustered standard errors on the class level following [21]; [] clustered standard errors on the class level using a bootstrapping method [20]; {} heteroscedastic robust unclustered standard errors. P-values below 0.1 in bold. Columns (1), (2) and (3) refer to the second assessment, columns (4), (5) and (6) to the follow-up.
(DOCX)

S4 Table Main outcome using ordered response models. Notes: Dependent variables are: choice (change in percentage of children choosing the dried apple), liking (change in liking measured on a 4-point scale), and comparison (change in number of times the dried apple is preferred in 5 pairwise comparisons). Independent variables are the binary Treatment-group indicator and baseline values are the respective food valuations in the first assessment; we also control for school specific fixed-effects. Estimates for choice based on a logit model, liking and comparison estimated by an ordered logit. P-values below the coefficients based on clustered standard errors on the class level. P-values below 0.1 in bold. Columns (1), (2) and (3) refer to the second assessment, columns (4), (5) and (6) to the follow-up.
(DOCX)

S5 Table Frequency and non-parametric significance test for change in valuation measures. Notes: Table provides the frequencies of change in the three outcome variables between baseline and the two following assessments. For each outcome variable we provide non-parametric tests whether changes are significantly different from zero (within-subject) and for the difference in the changes of valuation between control and treatment groups (between-subject). P-values below 0.1 in bold.
(DOCX)

S6 Table Estimation of exposure effect. *Notes*: Dependent variables are: choice (change in percentage of children choosing the dried apple), liking (change in liking measured on a 4-point scale), and comparison (change in number of times the dried apple is preferred in 5 pairwise comparisons). Independent variables a set of dummy variables indicating the assessment, with the 1^st^ assessment as reference. We also control for school specific fixed-effects. Estimates for choice based on a logit model, liking and comparison estimated by an ordered logit. P-values below the coefficients based on: () clustered standard errors on the individual level following [21]; {} heteroscedastic robust unclustered standard errors. P-values below 0.1 in bold.
(DOCX)

S7 Table Results from linear regression (standard errors computed with three alternative methods). Notes: Dependent variables are: Choice (change in percentage of children choosing the dried apple), Liking (change in liking measured on a 4-point scale), and Comparison (change in number of times the dried apple is preferred in 5 pairwise comparisons). Independent variables are: Reward (binary treatment-group indicator), Effort (binary indicator capturing the difference related to higher effort), Baseline value (the respective food valuations in the first assessment) and School (control for school specific fixed-effects). P-values below the coefficients based on: () clustered standard errors on the class level following [21]; [] clustered standard errors on the class level using a bootstrapping method [20]; {} heteroscedastic robust unclustered standard errors. P-values below 0.1 in bold. Columns (1), (2) and (3) refer to the second assessment, columns (4), (5) and (6) to the follow-up.
(DOCX)

S1 Appendix Survey study (reward use). (DOCX)

S2 Appendix Experimental materials. Experiment 1 (children): Child questionnaire (translated to English).
(DOCX)
