# Supplementary material for: Rewarding behavior with a sweet food strengthens its valuation
Source: PLoS One. 2021 Apr 14;16(4):e0242461. doi: 10.1371/journal.pone.0242461 (PMC8046216; doi:10.1371/journal.pone.0242461)
Supplement: S1 File — (DOCX) [file pone.0242461.s001.docx]

Dear Parent/children caretaker

We are a team of scientists from the University of Cologne and the University of Edinburgh. We would like to invite your child to take part in a research study on food preferences, that we intend to conduct at [NAME OF THE SCHOOL] on [DATES]. We would like to ask children to taste different foods and to indicate their liking of these foods. Furthermore, we would ask the children to solve simple tasks.

The study is part of a larger project funded by the European Union involving leading universities across Europe, which aims at understanding the drivers of food preferences at different ages. If you are interested in the details, you can visit our web page http://nudge-it.eu/. The project has been reviewed and approved by an academic ethical review committee, following the guidelines of the European Commission.

In case you have any questions about the project, please do not hesitate to contact us by telephone (0221-470-6939) or e-mail ([marina.schroeder@uni-koeln.de](mailto:marina.schroeder@uni-koeln.de)). In case your child has any dietary restriction (due to an allergy or an intolerance) or if you do not want your child to participate in the study, please fill in the attached sheet and hand it back to the class teacher by XXXXXX.

Thank you a lot in advance

Yours Faithfully,

**
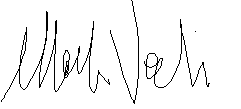
**
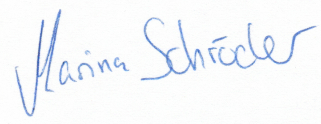

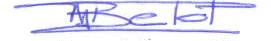


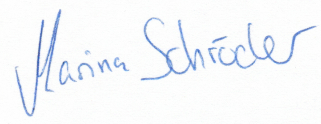


Prof. Dr. Michèle Belot Jun.-Prof. Dr. Marina Schröder Martina Vecchi

School of Economics Wirtschafts- und Sozial- School of Economics

University of Edinburgh wissenschaftliche Fakultät University of Edinburgh

Universität zu Köln

**Dietary restrictions**

Does your child have any **dietary restriction**?

⌊ Yes ⌋ ⌊ No⌋

Please specify below which foods you child **should not eat**:

___________________________________________________________________________

___________________________________________________________________________

___________________________________________________________________________

___________________________________________________________________________

**Exclusion of your child from this study**

- Tick the box beside if you **do not** want your child to participate to this research study.

**Name and surname of the child**

________________ ________________________________

Date Signature of parent/carer
