# Supplementary figures and images for: Rewarding behavior with a sweet food strengthens its valuation
Source: PLoS One. 2021 Apr 14;16(4):e0242461. doi: 10.1371/journal.pone.0242461 (PMC8046216; doi:10.1371/journal.pone.0242461)

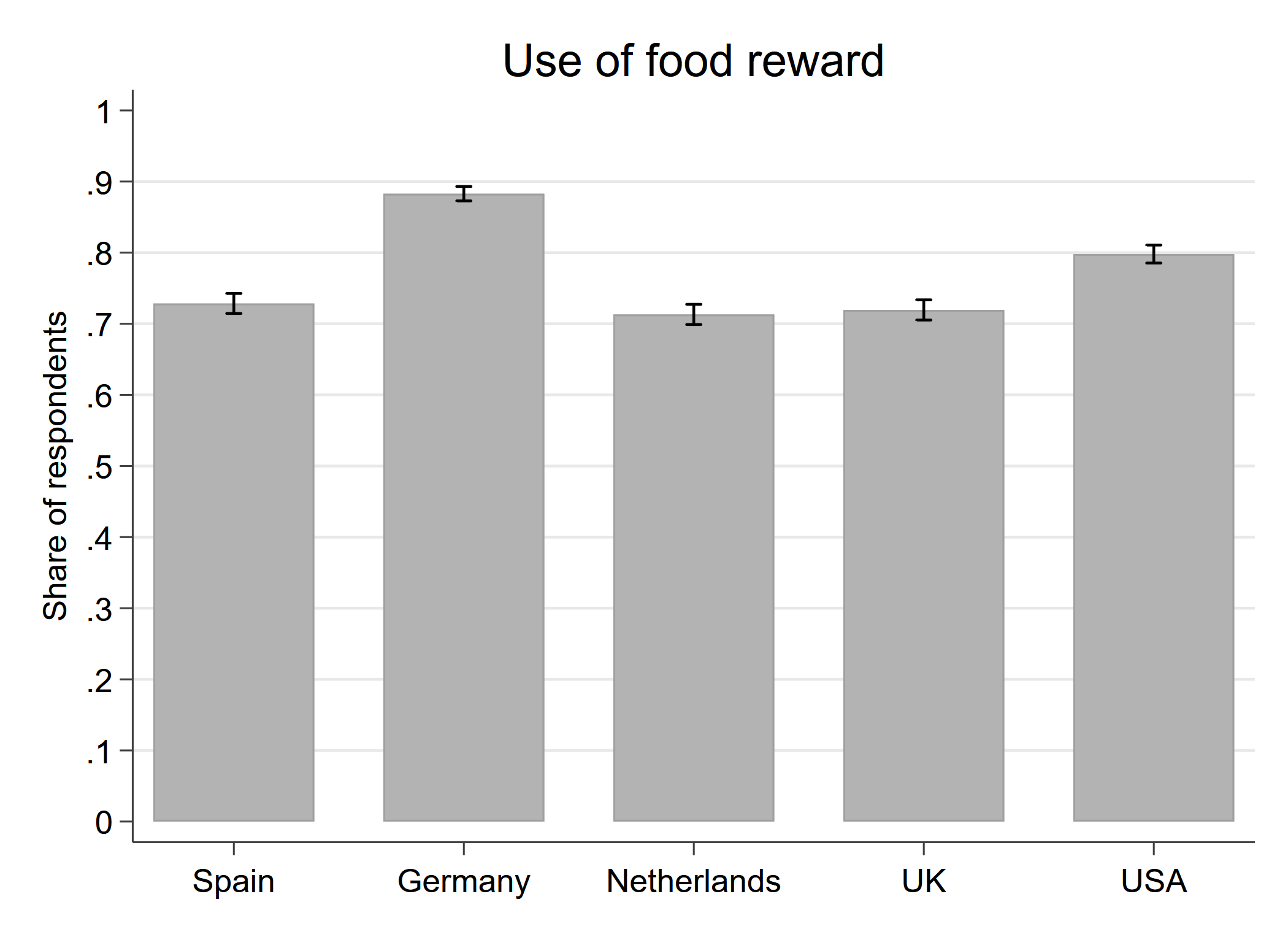

Supplement: S1 Fig — Share of participants reporting that in their family sweat treats are at least rarely used to reward for (1) doing something difficult, (2) doing something good or performing well, or (3) an achievement. Data are presented as mean ± 1 SEM. (TIF) [file pone.0242461.s002.tif]
