# Supplementary material for: Rewarding behavior with a sweet food strengthens its valuation
Source: PLoS One. 2021 Apr 14;16(4):e0242461. doi: 10.1371/journal.pone.0242461 (PMC8046216; doi:10.1371/journal.pone.0242461)
Supplement: S1 Table — a Values based on a parental survey response with varying sample size (N): Control (35); Low-Effort (48); High-effort (47). b Average frequency of attendance to the 6 treatment sessions. c Average frequency of trying the food reward to the 6 treatment sessions. d Average value at baseline. e P-values based on a two-sample Wilcoxon rank-sum (Mann-Whitney) test for ordinal variables. Fisher’s exact test is used for binary variables. (DOCX) [file pone.0242461.s003.docx]

**S1 Table. Summary Statistics and Initial Levels.**

|  | (1) | (2) | (3) | (4) | (5) | (6) | (7) | (8) |
| --- | --- | --- | --- | --- | --- | --- | --- | --- |
|  | Control | Low effort | High effort | Total | Min | Max | (1)vs.(2)^e^ | (1)vs.(3)^e^ |
| Number of children: |  |  |  |  |  |  |  |  |
| total | 59 | 79 | 76 | 214 |  |  |  |  |
| allergic to apples | 1 | 0 | 1 | 2 |  |  |  |  |
| incomplete data | 10 | 11 | 14 | 35 |  |  |  |  |
| analyzed | 48 | 68 | 61 | 177 |  |  |  |  |
| Number of classes | 3 | 4 | 4 | 11 |  |  |  |  |
| Age (years)^a^ | 6.59 | 6.59 | 6.68 | 6.62 | 6 | 8 | 0.78 | 0.50 |
| Female (%)^a^ | 45 | 41 | 47 | 44 | 0 | 1 | 0.66 | 0.84 |
| Attendance^b^ | 5.79 | 5.62 | 5.57 | 5.65 | 0 | 6 | 0.11 | 0.24 |
| Tried reward^c^ | 4.13 | 4.16 | 3.98 | 4.09 | 0 | 6 | 0.92 | 0.80 |
| Choice^d^ | 0.09 | 0.06 | 0.03 | 0.06 | 0 | 1 | 0.61 | 0.25 |
| Liking^d^ | 1.31 | 1.57 | 1.26 | 1.4 | 0 | 3 | 0.32 | 0.96 |
| Comparison^d^ | 0.94 | 0.79 | 0.90 | 0.87 | 0 | 5 | 0.53 | 0.77 |
| NoNotes: ^a^ Values based on a parental survey response with varying sample size (N). Lowest of age: Control (35); Low-Effort (49); High-effort (47). ^b^ Average frequency of attendance to the 6 treatment sessions. ^c^ Average frequency of trying the food reward to the 6 treatment sessions. ^d^ Average value at baseline. ^e^ P-values based on a two-sample Wilcoxon rank-sum (Mann-Whitney) test for ordinal variables. Chi-squared test is used for binary variables. | | | | | | | | |
