# Supplementary material for: Rewarding behavior with a sweet food strengthens its valuation
Source: PLoS One. 2021 Apr 14;16(4):e0242461. doi: 10.1371/journal.pone.0242461 (PMC8046216; doi:10.1371/journal.pone.0242461)
Supplement: S2 Table — Notes: Linear probability model predicting missing values in the data. P-values below 0.1 in bold based on clustered on the class level in parenthesis. Binary dependent variable = 1 if at least 1 of 3 outcomes is missing during at least one assessment. (DOCX) [file pone.0242461.s004.docx]

**S2 Table. Attrition.**

|  | (1) | (2) | (3) | (4) |
| --- | --- | --- | --- | --- |
|  | Attrition | Attrition | Attrition | Attrition |
| Control | *reference* | | | |
| Low effort | -0.0472 |  |  |  |
|  | (0.316) |  |  |  |
| High effort | 0.0109 |  |  |  |
|  | (0.655) |  |  |  |
| Choice |  | 0.0275 |  |  |
|  |  | (0.841) |  |  |
| Liking |  |  | -0.0133 |  |
|  |  |  | (0.305) |  |
| Comparison |  |  |  | 0.0215 |
|  |  |  |  | (0.454) |
| Constant | 0.186 | 0.139 | 0.160 | 0.118 |
|  | **(0.000)** | **(0.000)** | **(0.003)** | **(0.007)** |
| N | 214 | 206 | 203 | 205 |
| NoNotes: OLS predicting missing values in the data. P-values < 0.1 in bold, based on clustered on the class level in parenthesis [21]. Binary dependent variable =1 if at least 1 of 3 outcomes is missing during at least one assessment. | | | | |
