# Supplementary material for: Rewarding behavior with a sweet food strengthens its valuation
Source: PLoS One. 2021 Apr 14;16(4):e0242461. doi: 10.1371/journal.pone.0242461 (PMC8046216; doi:10.1371/journal.pone.0242461)
Supplement: S3 Table — Notes: Dependent variables are: choice (change in percentage of children choosing the dried apple), liking (change in liking measured on a 4-point scale), and comparison (change in number of times the dried apple is preferred in 5 pairwise comparisons). Independent variables are the binary Treatment-group indicator and baseline values are the respective food valuations in the first assessment; we also control for school specific fixed-effects. Model based on OLS. P-values below the coefficients based on: () clustered standard errors on the class level following [21]; [] clustered standard errors on the class level using a bootstrapping method [20]; {} heteroscedastic robust unclustered standard errors. P-values below 0.1 in bold. Columns (1), (2) and (3) refer to the second assessment, columns (4), (5) and (6) to the follow-up. (DOCX) [file pone.0242461.s005.docx]

**S3 Table. Main outcome**

|  | (1) | (2) | (3) |  | (4) | (5) | (6) |
| --- | --- | --- | --- | --- | --- | --- | --- |
|  | Short-term | | |  | Long-term | | |
|  | *Choice* | *Liking* | *Comparison* |  | *Choice* | *Liking* | *Comparison* |
| Reward | 0.0753 | 0.283 | 0.451 |  | 0.136 | -0.154 | 0.479 |
|  | **(0.014)** | **(0.020)** | **(0.005)** |  | **(0.003)** | (0.456) | **(0.000)** |
|  | **[0.044]** | **[0.056]** | **[0.018]** |  | **[0.012]** | [0.57] | **[<0.001]** |
|  | {0.140} | **{0.084}** | **{0.004}** |  | **{0.042}** | {0.386} | **{0.005}** |
| Baseline value | 0.542 | 0.610 | 0.490 |  | 0.136 | 0.515 | 0.522 |
|  | **(0.002)** | **(0.000)** | **(0.002)** |  | **(0.003)** | **(0.000)** | **(0.000)** |
|  | **[<0.001]** | **[<0.001]** | **[0.002]** |  | **[<0.001]** | **[<0.001]** | **[<0.001]** |
|  | **{<0.001}** | **{<0.001}** | **{<0.001}** |  | **{0.008}** | **{<0.001}** | **{<0.001}** |
| School 1 | ref. | ref. | ref. |  | ref. | ref. | ref. |
| School 2 | -0.115 | 0.149 | 0.328 |  | -0.00682 | 0.0624 | 0.433 |
|  | **(0.038)** | (0.264) | **(0.087)** |  | (0.909) | (0.808) | **(0.023)** |
|  | **[0.214]** | [0.404] | **[0.18]** |  | [0.84] | [0.778] | **[0.208]** |
|  | **{0.066}** | {0.508} | {0.108} |  | {0.937} | {0.781} | **{0.080}** |
| School 3 | -0.0832 | 0.174 | 0.127 |  | -0.0663 | 0.488 | 0.211 |
|  | (0.109) | (0.131) | (0.355) |  | (0.384) | **(0.089)** | (0.296) |
|  | [0.370] | [0.184] | [0.420] |  | [0.560] | [0.260] | [0.446] |
|  | {0.197} | {0.399} | {0.506} |  | {0.388} | **{0.021}** | {0.314} |
| Constant | 0.110 | 1.205 | 0.663 |  | 0.136 | 1.777 | 0.919 |
|  | **(0.016)** | **(0.000)** | **(0.001)** |  | **(0.019)** | **(0.001)** | **(0.000)** |
|  | [0.246] | **[<0.001]** | **[<0.001]** |  | **[<0.001]** | **[0.064]** | **[<0.001]** |
|  | **{0.059}** | **{<0.001}** | **{<0.001}** |  | **{0.064}** | **{<0.001}** | **{<0.001}** |
| *N* | 177 | 177 | 177 |  | 177 | 177 | 177 |
| Notes: Dependent variables are: choice (dummy for children choosing the dried apple), liking (liking measured on a 4-point scale), and comparison (number of times the dried apple is preferred in 5 pairwise comparisons). Independent variables are the binary treatment-group indicator and baseline values are the respective food valuations in the first assessment; we also control for school specific fixed-effects. Models estimated via OLS. P-values below the coefficients based on: () clustered standard errors on the class level following [21]; [] clustered standard errors on the class level using a bootstrapping method [20]; {} heteroscedastic robust unclustered standard errors. P-values < 0.1 in bold. Columns (1), (2) and (3) refer to the second assessment, columns (4), (5) and (6) to the third assessment. | | | | | | | |
