# Supplementary material for: Rewarding behavior with a sweet food strengthens its valuation
Source: PLoS One. 2021 Apr 14;16(4):e0242461. doi: 10.1371/journal.pone.0242461 (PMC8046216; doi:10.1371/journal.pone.0242461)
Supplement: S4 Table — Notes: Dependent variables are: choice (change in percentage of children choosing the dried apple), liking (change in liking measured on a 4-point scale), and comparison (change in number of times the dried apple is preferred in 5 pairwise comparisons). Independent variables are the binary Treatment-group indicator and baseline values are the respective food valuations in the first assessment; we also control for school specific fixed-effects. Estimates for choice based on a logit model, liking and comparison estimated by an ordered logit. P-values below the coefficients based on clustered standard errors on the class level. P-values below 0.1 in bold. Columns (1), (2) and (3) refer to the second assessment, columns (4), (5) and (6) to the follow-up. (DOCX) [file pone.0242461.s006.docx]

**S4 Table.** **Main outcome using ordered response models.**

|  | (1) | (2) | (3) |  | (4) | (5) | (6) |
| --- | --- | --- | --- | --- | --- | --- | --- |
|  | Short-term | | |  | Long-term | | |
|  | *Choice* | *Liking* | *Comparison* |  | *Choice* | *Liking* | *Comparison* |
| Reward | 2.495 | 2.440 | 2.067 |  | 2.495 | 0.695 | 1.710 |
|  | **(<0.001)** | **(<0.001)** | **(0.010)** |  | **(<0.001)** | (0.449) | **(0.003)** |
| Baseline value | 27.94 | 3.599 | 2.708 |  | 6.531 | 3.004 | 2.196 |
|  | **(<0.001)** | **(<0.001)** | **(<0.001)** |  | **(<0.001)** | **(<0.001)** | **(<0.001)** |
| School 1 | ref. | ref. | ref. |  | ref. | ref. | ref. |
| School 2 | 0.274 | 1.247 | 1.824 |  | 0.964 | 1.383 | 1.754 |
|  | **(0.013)** | (0.406) | **(0.099)** |  | (0.913) | (0.549) | **(0.034)** |
| School 3 | 0.456 | 1.271 | 1.212 |  | 0.672 | 3.114 | 1.311 |
|  | **(0.047)** | (0.385) | (0.420) |  | (0.356) | **(0.072)** | (0.376) |
| Constant | 0.0946 |  |  |  | 0.150 |  |  |
|  | **(<0.001)** |  |  |  | **(<0.001)** |  |  |
| Cut 1 |  | 2.502 | 0.624 |  |  | 1.113 | 0.402 |
|  |  | **(0.006)** | (0.124) |  |  | (0.871) | **(0.017)** |
| Cut 2 |  | 3.953 | 6.589 |  |  | 1.689 | 2.590 |
|  |  | **(<0.001)** | **(<0.001)** |  |  | **(0.401)** | **(<0.001)** |
| Cut 3 |  | 8.012 | 32.60 |  |  | 2.733 | 11.15 |
|  |  | **(<0.001)** | **(<0.001)** |  |  | **(0.151)** | **(<0.001)** |
| Cut 4 |  |  | 106.2 |  |  |  | 30.61 |
|  |  |  | **(<0.001)** |  |  |  | **(<0.001)** |
| Cut 5 |  |  | 661.0 |  |  |  | 90.85 |
|  |  |  | **(<0.001)** |  |  |  | **(<0.001)** |
| *N* | 177 | 177 | 177 |  | 177 | 177 | 177 |
| Notes: Dependent variables are: choice (dummy for children choosing the dried apple), liking (liking measured on a 4-point scale), and comparison (number of times the dried apple is preferred in 5 pairwise comparisons). Independent variables are the binary treatment-group indicator and baseline values are the respective food valuations in the first assessment; we also control for school specific fixed-effects. Estimates for choice based on a logit model, liking and comparison estimated by an ordered logit. P-values below the coefficients based on clustered standard errors on the class level following [21]. P-values < 0.1 in bold. Columns (1), (2) and (3) refer to the second assessment, columns (4), (5) and (6) to the third assessment. | | | | | | | |
