# Supplementary material for: Rewarding behavior with a sweet food strengthens its valuation
Source: PLoS One. 2021 Apr 14;16(4):e0242461. doi: 10.1371/journal.pone.0242461 (PMC8046216; doi:10.1371/journal.pone.0242461)
Supplement: S5 Table — Notes: Table provides the frequencies of change in the three outcome variables between baseline and the two following assessments. For each outcome variable we provide non-parametric tests whether changes are significantly different from zero (within-subject) and for the difference in the changes of valuation between control and treatment groups (between-subject). P-values below 0.1 in bold. (DOCX) [file pone.0242461.s007.docx]

**S5 Table. Frequency and non-parametric significance tests for differences in valuation measures.**

|  | (1) | (2) |  | (3) | (4) |
| --- | --- | --- | --- | --- | --- |
|  | Change between  1^st^ & 2^nd^ assessment | |  | Change between  1^st^ & 3^rd^ assessment | |
|  | Control | Treatment | | Control | Treatment |
| *Δ choice (frequency)* |  |  | |  |  |
| -1 | 3 | 1 |  | 3 | 1 |
| 0 | 42 | 115 |  | 39 | 100 |
| 1 | 3 | 13 |  | 6 | 28 |
| Wilcoxon signed-rank test: H0 *Δ choice* = 0 | | | | | |
| z | 2.593 | 3.207 |  | 1 | 5.014 |
| P-value | **(0.006)** | **(0.002)** |  | (0.508) | **(<0.001)** |
| Chi2 test: H0 *Δ choice* control = *Δ choice* treatment | | | | | |
| chi2 |  | 5.218 |  |  | 6.245 |
| P-value |  | **(0.074)** |  |  | **(0.044)** |
| *Δ liking (frequency)* |  |  |  |  |  |
| -3 | 0 | 1 |  | 1 | 0 |
| -2 | 0 | 3 |  | 0 | 2 |
| -1 | 2 | 3 |  | 0 | 8 |
| 0 | 35 | 69 |  | 25 | 68 |
| 1 | 3 | 22 |  | 9 | 17 |
| 2 | 5 | 17 |  | 5 | 16 |
| 3 | 3 | 14 |  | 8 | 18 |
| Wilcoxon signed-rank test: H0 *Δ liking* = 0 | | | | | |
| z | 2.593 | 5.9 |  | 4.241 | 5.527 |
| P-value | **(0.006)** | **(<0.001)** |  | **(<0.001)** | **(<0.001)** |
| U- test: H0 *Δ liking* control = *Δ liking* treatment | | | | | |
| z |  | 1.754 |  |  | 0.923 |
| P-value |  | **(0.0776)** |  |  | (0.3567) |
| *Δ comparison (frequency)* |  |  |  |  |  |
| -5 | 0 | 0 |  | 0 | 0 |
| -4 | 1 | 0 |  | 0 | 0 |
| -3 | 0 | 0 |  | 0 | 0 |
| -2 | 1 | 0 |  | 1 | 0 |
| -1 | 3 | 6 |  | 2 | 8 |
| 0 | 22 | 55 |  | 19 | 38 |
| 1 | 17 | 37 |  | 16 | 36 |
| 2 | 3 | 18 |  | 8 | 22 |
| 3 | 1 | 12 |  | 2 | 18 |
| 4 | 0 | 1 |  | 0 | 5 |
| 5 | 0 | 0 |  | 0 | 2 |
| Wilcoxon signed-rank test: H0 *Δ comparison* = 0 | | | | | |
| z | 2.964 | 7.29 |  | 4.211 | 8 |
| P-value | **(0.003)** | **(<0.001)** |  | **(<0.001)** | **(<0.001)** |
| U- test: H0 *Δ comparison* control = *Δ comparison* treatment | | | | | |
| z |  | 1.946 |  |  | 1.983 |
| P-value |  | **(0.051)** |  |  | **(0.047)** |
| Notes: Table provides the frequencies of change in the three outcome variables between baseline and the two following assessments. For each outcome variable we provide non-parametric tests whether changes are significantly different from zero (within-subject) and for the difference in the changes of valuation between control and treatment groups (between-subject). P-values < 0.1 in bold. | | | | | |
