# Supplementary material for: Rewarding behavior with a sweet food strengthens its valuation
Source: PLoS One. 2021 Apr 14;16(4):e0242461. doi: 10.1371/journal.pone.0242461 (PMC8046216; doi:10.1371/journal.pone.0242461)
Supplement: S6 Table — Notes: Dependent variables are: choice (change in percentage of children choosing the dried apple), liking (change in liking measured on a 4-point scale), and comparison (change in number of times the dried apple is preferred in 5 pairwise comparisons). Independent variables a set of dummy variables indicating the assessment, with the 1st assessment as reference. We also control for school specific fixed-effects. Estimates for choice based on a logit model, liking and comparison estimated by an ordered logit. P-values below the coefficients based on: () clustered standard errors on the individual level following [21]; {} heteroscedastic robust unclustered standard errors. P-values below 0.1 in bold. (DOCX) [file pone.0242461.s008.docx]

**S6 Table. Estimation of exposure effect.**

|  | (1) | (2) | (3) |
| --- | --- | --- | --- |
| Model | Logit | Ordered logit | Ordered logit |
| Dependent variable | Choice | Valuation | Comparison |
| 1^st^ assessment | Ref. | Ref. | Ref. |
|  |  |  |  |
| 2nd assessment | 1.0000 | 1.7027 | 2.3021 |
|  | (1.000) | **(0.009)** | **(0.005)** |
|  | {1.000} | {0.164} | **{0.029}** |
| 3rd assessment | 1.8906 | 3.4678 | 4.9789 |
|  | (0.321) | **(<0.001)** | **(<0.001)** |
|  | {0.337} | **{0.003}** | **{<0.001}** |
| School 1 | Ref. | Ref. | Ref. |
|  |  |  |  |
| School 2 | 2.1375 | 1.8512 | 0.5280 |
|  | (0.399) | (0.321) | (0.227) |
|  | {0.285} | {0.126} | **{0.096}** |
| School 3 | 1.2706 | 1.2144 | 0.5908 |
|  | (0.808) | (0.723) | (0.413) |
|  | {0.764} | {0.600} | {0.224} |
| Constant | 0.0598 |  |  |
|  | **(<0.001)** |  |  |
|  | **{<0.001}** |  |  |
|  |  |  |  |
| Cut 1 |  | 1.0711 | 0.3811 |
|  |  | (0.877) | **(0.075)** |
|  |  | {0.848} | **{0.022}** |
| Cut 2 |  | 1.6192 | 2.1748 |
|  |  | (0.284) | (0.103) |
|  |  | {0.175} | **{0.047}** |
| Cut 3 |  | 2.8760 | 20.5635 |
|  |  | **(0.018)** | **(<0.001)** |
|  |  | **{0.004}** | **{<0.001}** |
| Cut 4 |  |  | 88.9768 |
|  |  |  | **(<0.001)** |
|  |  |  | **{<0.001}** |
| *N* | 48 | 48 | 48 |
| *Obs.* | 144 | 144 | 144 |
| *Notes:* Dependent variables are: choice (dummy for children choosing the dried apple), liking (liking measured on a 4-point scale), and comparison (number of times the dried apple is preferred in 5 pairwise comparisons).. Independent variables a set of dummy variables indicating the assessment, with the 1^st^ assessment as reference. We also control for school specific fixed-effects. Estimates for choice based on a logit model, liking and comparison estimated by an ordered logit. P-values below the coefficients based on; () clustered standard errors on the individual level following [21]; {} heteroscedastic robust unclustered standard errors. P-values < 0.1 in bold. | | | |
