# Supplementary material for: Rewarding behavior with a sweet food strengthens its valuation
Source: PLoS One. 2021 Apr 14;16(4):e0242461. doi: 10.1371/journal.pone.0242461 (PMC8046216; doi:10.1371/journal.pone.0242461)
Supplement: S7 Table — Notes: Dependent variables are: Choice (change in percentage of children choosing the dried apple), Liking (change in liking measured on a 4-point scale), and Comparison (change in number of times the dried apple is preferred in 5 pairwise comparisons). Independent variables are: Reward (binary treatment-group indicator), Effort (binary indicator capturing the difference related to higher effort), Baseline value (the respective food valuations in the first assessment) and School (control for school specific fixed-effects). P-values below the coefficients based on: () clustered standard errors on the class level following [21]; [] clustered standard errors on the class level using a bootstrapping method [20]; {} heteroscedastic robust unclustered standard errors. P-values below 0.1 in bold. Columns (1), (2) and (3) refer to the second assessment, columns (4), (5) and (6) to the follow-up. (DOCX) [file pone.0242461.s009.docx]

**S7 Table. Difference between two treatment groups.**

|  | (1) | (2) | (3) |  | (4) | (5) | (6) |
| --- | --- | --- | --- | --- | --- | --- | --- |
|  | Short-term | | |  | Long-term | | |
|  | *Choice* | *Liking* | *Comparison* |  | *Choice* | *Liking* | *Comparison* |
| Reward | 0.0883 | 0.297 | 0.567 |  | 0.123 | -0.0624 | 0.543 |
|  | **(0.011)** | **(0.056)** | **(0.001)** |  | **(0.021)** | (0.795) | **(<0.001)** |
|  | **[0.054]** | [0.182] | **[0.030]** |  | **[0.062]** | [0.806] | **[<0.001]** |
|  | {0.140} | {0.107} | **{0.002}** |  | {0.103} | {0.748} | **{0.007}** |
| High effort | -0.0282 | -0.0301 | -0.250 |  | 0.0285 | -0.195 | -0.139 |
|  | (0.455) | (0.838) | **(0.065)** |  | (0.658) | (0.281) | (0.375) |
|  | [0.192] | [0.842] | [0.132] |  | [0.722] | [0.422] | [0.492] |
|  | {0.599} | {0.874} | {0.173} |  | {0.700} | {0.316} | {0.558} |
| Baseline value | 0.540 | 0.609 | 0.496 |  | 0.403 | 0.509 | 0.526 |
|  | **(0.002)** | **(<0.001)** | **(0.001)** |  | **(0.005)** | **(<0.001)** | **(<0.001)** |
|  | **[<0.001]** | **[<0.001]** | **[<0.001]** |  | **[<0.001]** | **[<0.001]** | **[<0.001]** |
|  | **{<0.001}** | **{<0.001}** | **{<0.001}** |  | **{0.008}** | **{<0.001}** | **{<0.001}** |
| School 1 | ref. | ref. | ref. |  | ref. | ref. | ref. |
| School 2 | -0.114 | 0.150 | 0.338 |  | -0.00807 | 0.0694 | 0.438 |
|  | **(0.020)** | (0.276) | **(0.017)** |  | (0.904) | (0.759) | **(0.010)** |
|  | [0.122] | [0.456] | **[0.078]** |  | [0.810] | [0.752] | **[0.060]** |
|  | {0.068} | {0.504} | **{0.094}** |  | {0.925} | {0.757} | **{0.075}** |
| School 3 | -0.0809 | 0.177 | 0.147 |  | -0.0686 | 0.504 | 0.222 |
|  | **(0.081)** | (0.110) | (0.193) |  | (0.405) | **(0.053)** | (0.200) |
|  | [0.274] | [0.166] | [0.284] |  | [0.598] | [0.174] | [0.408] |
|  | {0.202} | {0.393} | {0.439} |  | {0.367} | **{0.017}** | {0.291} |
| Constant | 0.109 | 1.206 | 0.647 |  | 0.137 | 1.785 | 0.910 |
|  | **(0.009)** | **(<0.001)** | **(<0.001)** |  | **(0.029)** | **(<0.001)** | **(<0.001)** |
|  | [0.212] | **[<0.001]** | **[<0.001]** |  | **[<0.001]** | **[0.008]** | **[<0.001]** |
|  | **{0.061}** | **{<0.001}** | **{<0.001}** |  | **{0.062}** | **{<0.001}** | **{<0.001}** |
| *N* | 177 | 177 | 177 |  | 177 | 177 | 177 |
| Notes: Dependent variables are: choice (dummy for children choosing the dried apple), liking (liking measured on a 4-point scale), and comparison (number of times the dried apple is preferred in 5 pairwise comparisons). Independent variables are: Reward (binary treatment-group indicator), Effort (binary indicator capturing the difference related to higher effort), Baseline value (the respective food valuations in the first assessment) and School (control for school specific fixed-effects). P-values below the coefficients based on: () clustered standard errors on the class level following [21]; [] clustered standard errors on the class level using a bootstrapping method [20]; {} heteroscedastic robust unclustered standard errors. P-values < 0.1 in bold. Columns (1), (2) and (3) refer to the second assessment, columns (4), (5) and (6) to the follow-up. | | | | | | | |
