# Supplementary material for: Rewarding behavior with a sweet food strengthens its valuation
Source: PLoS One. 2021 Apr 14;16(4):e0242461. doi: 10.1371/journal.pone.0242461 (PMC8046216; doi:10.1371/journal.pone.0242461)
Supplement: S1 Appendix — (DOCX) [file pone.0242461.s010.docx]

**S1 Appendix. Survey study (reward use)**

The data collection targeted five countries with different food cultures, namely: Spain, Germany, Netherlands, the U.K. and the U.S. Within each country we aimed for a target sample of 1,000 participants. The target sample ranged from 18 to 80 years. The survey was administered online and aimed to represent the general population. We applied a stratified sampling strategy using census-based quotas for age, gender and region (NUTS1 for Europe, 4 regions in the U.S.).

The data collection was facilitated via a panel provider (i.e. www.lightspeedresearch.com) and the survey design was using Qualtrics (www.qualtrics.com). For each country, participants in the target sample were invited to participate in the survey to receive a reward. They were screened and could start the survey only if they (i) provided informed consent and (ii) fitted the remaining quota criteria. As quality measures, the survey excluded participants if they (i) wrongly answered a filter question, (ii) needed less than 5 minutes to finish the survey (a threshold decided upon pre-testing), or (iii) provided illogical responses (e.g. making 0 food choices on an average day). These excluded participants were resampled as far as possible.

The survey items for the reward use were designed in English and translated into the respective languages. The items were checked by a second translator and discrepancies were resolved. The English version asks participants: “Under what circumstances would a child in your household or family receive a “sweet treat”?”. Participants responded to seven questions from which three are used in the analysis (i.e. As a reward for doing something difficult; As a reward for doing something good or performing well; As a reward for an achievement). The other four questions were mainly included to distract from the purpose of the questions [i.e. When they ask for it; In social settings and parties (such as birthday parties); As a compensation when they feel sad or stressed; As a reward for finishing a meal]. The five-item measurement scale ranged from 4 “Very frequently” to 0 “Never.”

Data was analyzed with STATA 16. The deviation from the pre-defined target sample was below 1% and we hence did not apply survey weights to the data. Final sample sizes were: Spain n=1,002, Germany n= 1,008, Netherlands n= 1,018, U.K. n=998, U.S. n= 1,010. Most families (71.88%) reports to use food as reward. Country-specific statistics provided in S1 Fig. are calculated based on participants, who at least respond with 1 “Rarely” to one of the reward questions.
