# Supplementary material for: Rewarding behavior with a sweet food strengthens its valuation
Source: PLoS One. 2021 Apr 14;16(4):e0242461. doi: 10.1371/journal.pone.0242461 (PMC8046216; doi:10.1371/journal.pone.0242461)
Supplement: S2 Appendix — Experiment 1 (children): Child questionnaire (translated to English). (DOCX) [file pone.0242461.s011.docx]

S2 Appendix. Experimental materials.

Experiment 1 (children): Child questionnaire (translated to English)


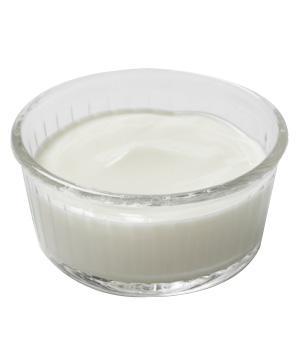

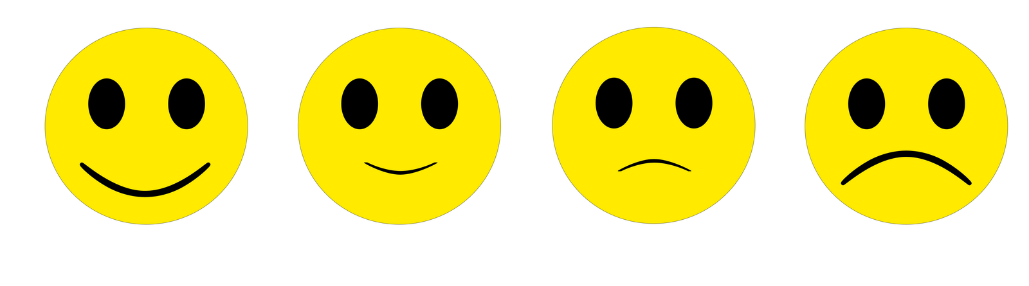


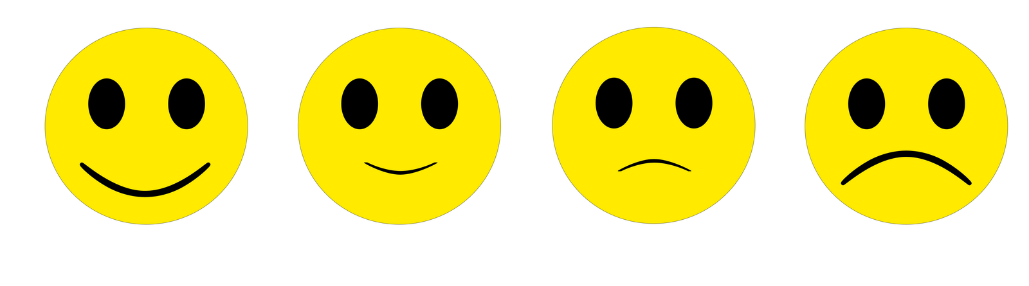

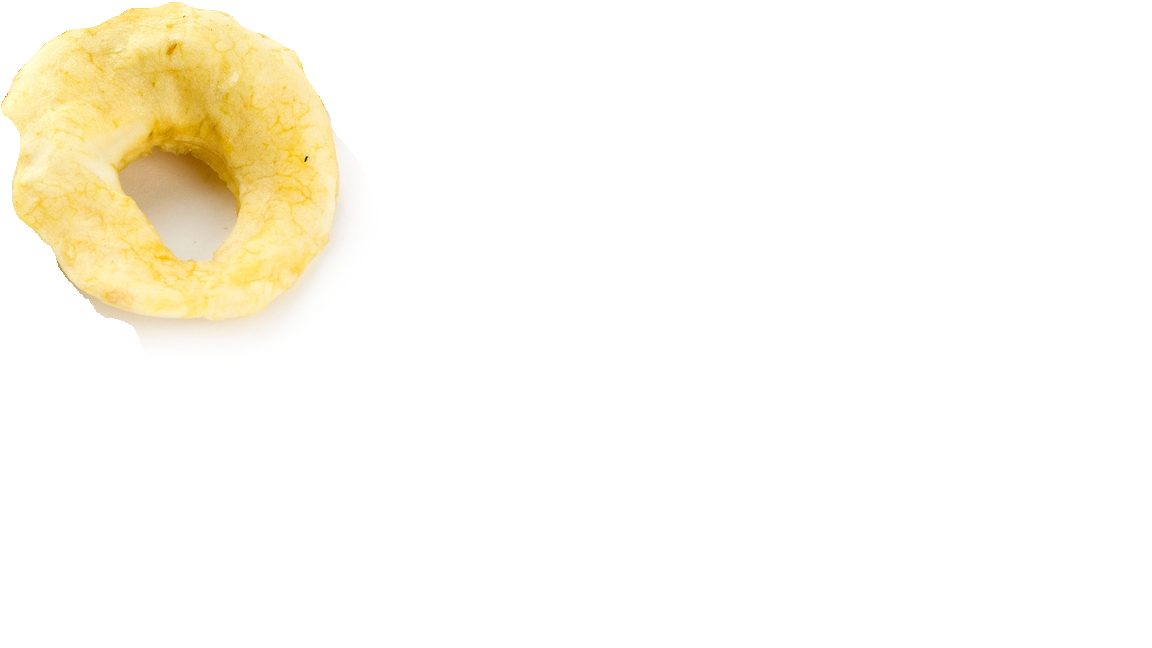

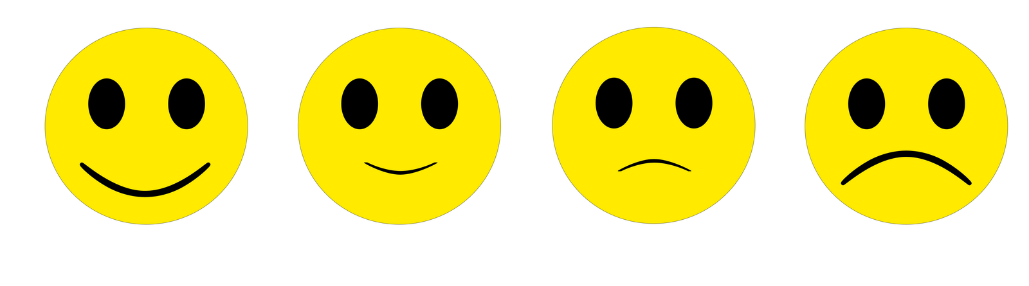

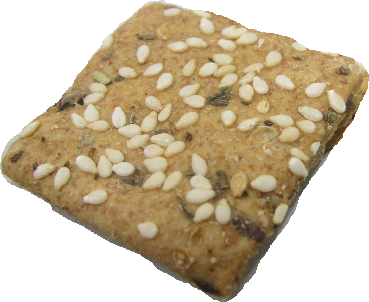

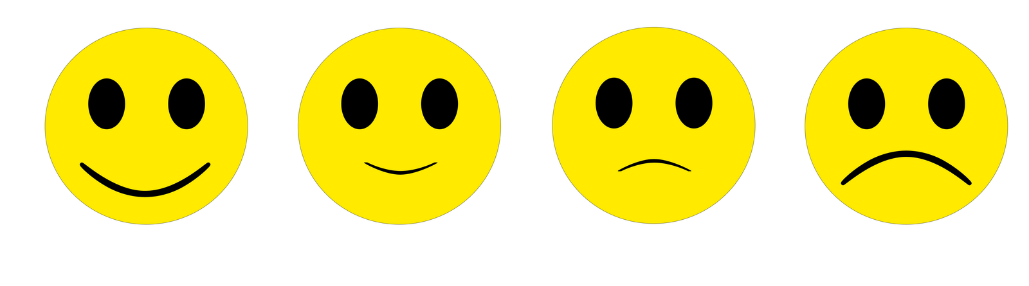

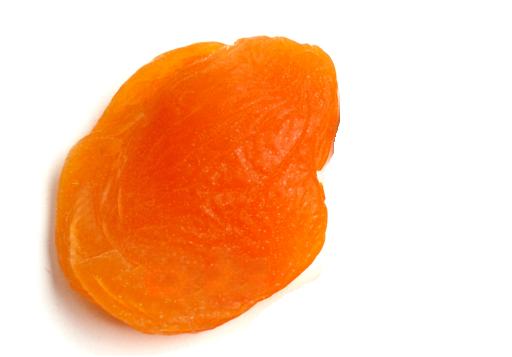


**Which food would you would like to receive as a snack
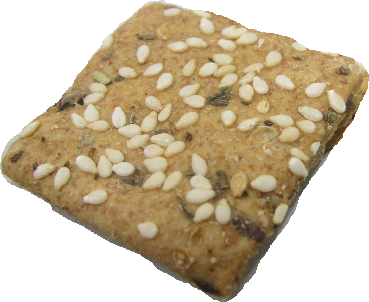

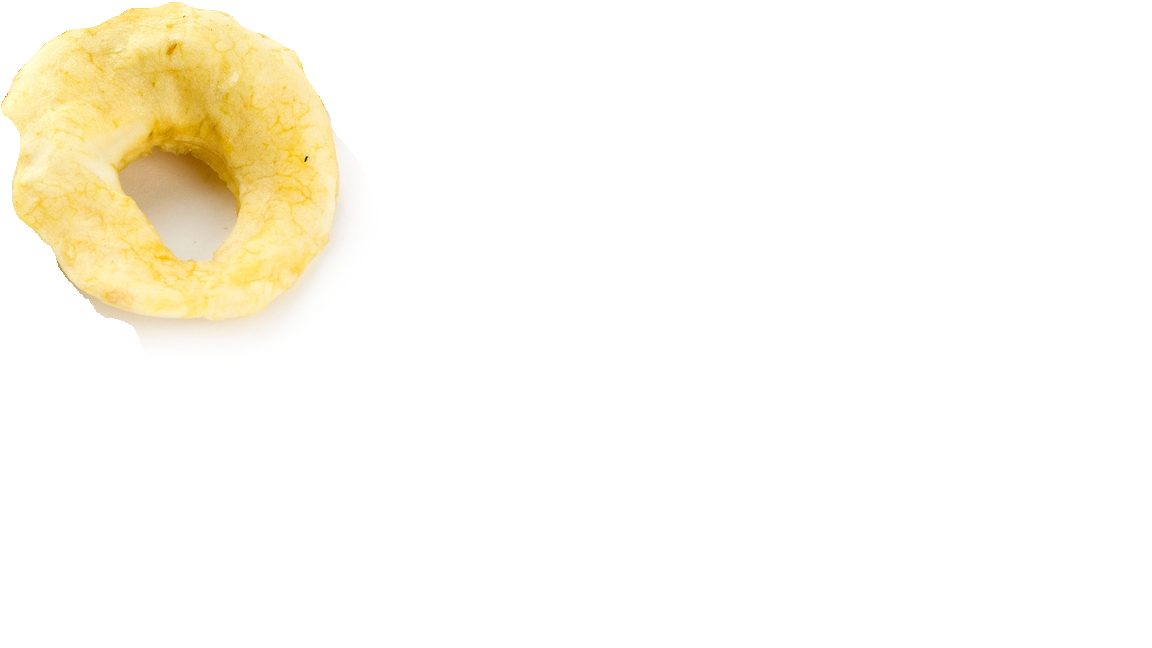

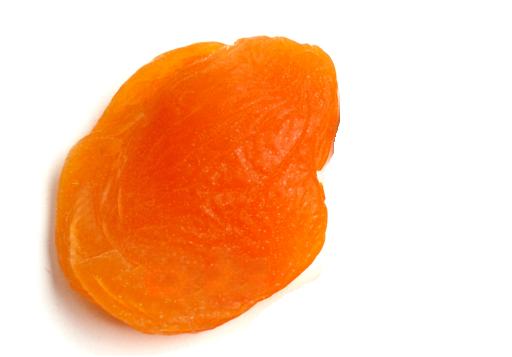

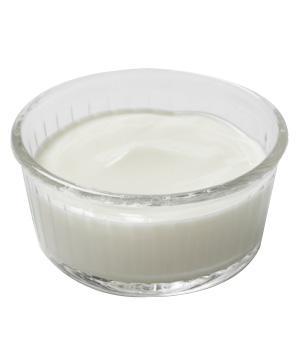
?**

QUESTION 1

| Dried apple  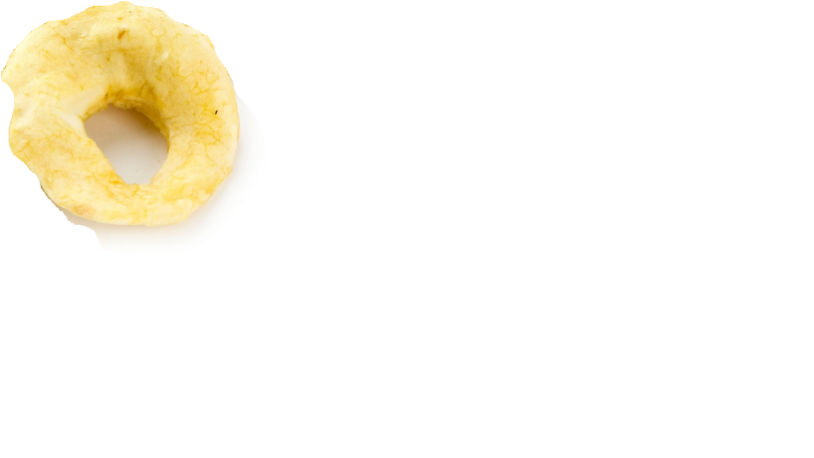 | French fry  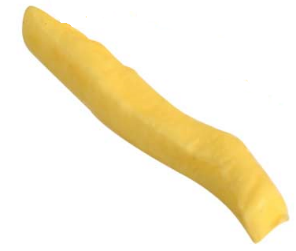 |
| --- | --- |

What would you prefer to eat?

QUESTION 2

| Dried apricot  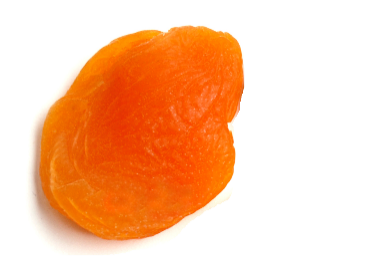 | Dried apple  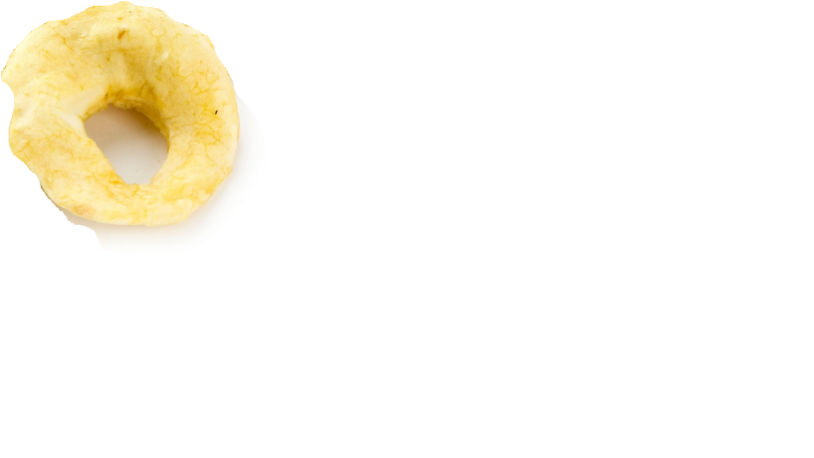 |
| --- | --- |

What would you prefer to eat?

QUESTION 3

| Natural yoghurt  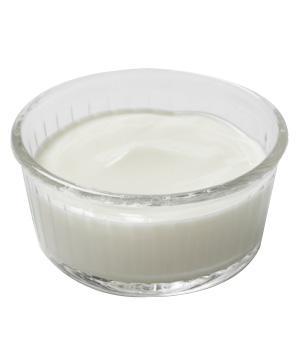 | Wholegrain cracker  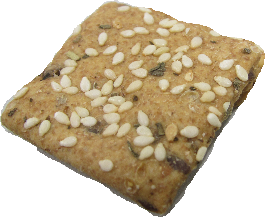 |
| --- | --- |

What would you prefer to eat?

QUESTION 4

| Dried apricot  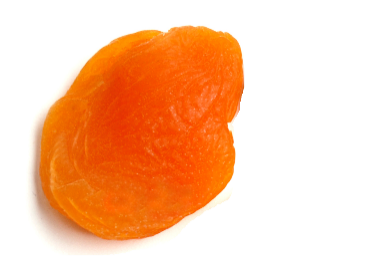 | Natural yoghurt  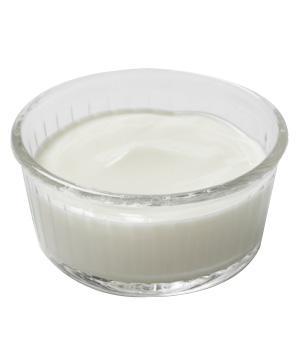 |
| --- | --- |

What would you prefer to eat?

QUESTION 5

| Dried Apple  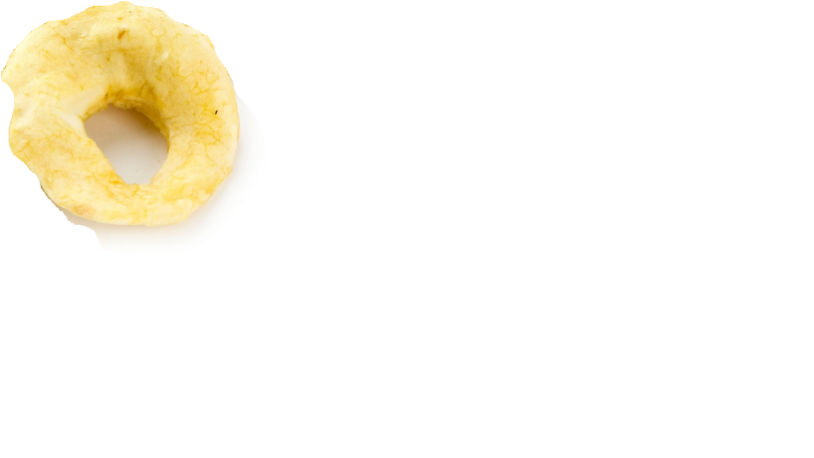 | Natural yoghurt  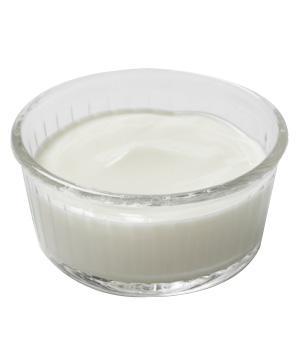 |
| --- | --- |

What would you prefer to eat?

QUESTION 6

| Wholegrain cracker  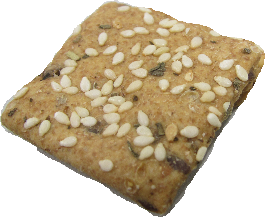 | Dried apple  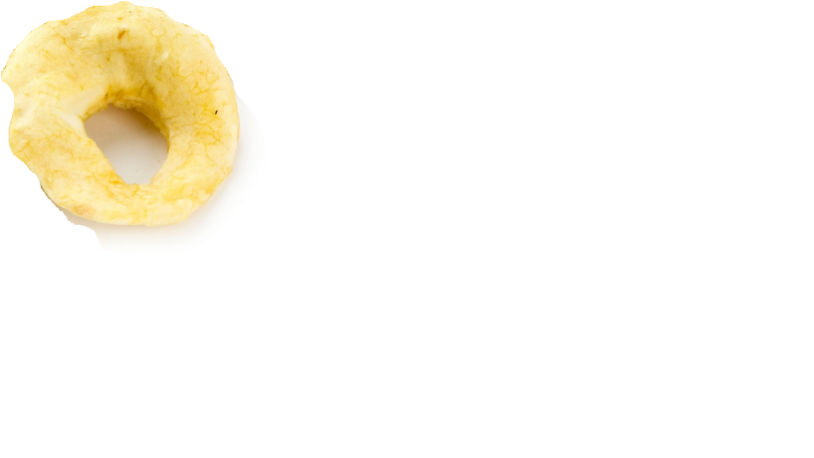 |
| --- | --- |

What would you prefer to eat?

QUESTION 7

| Gummy bear  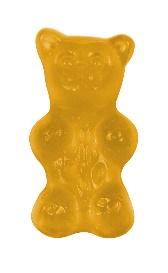 | Dried Apple  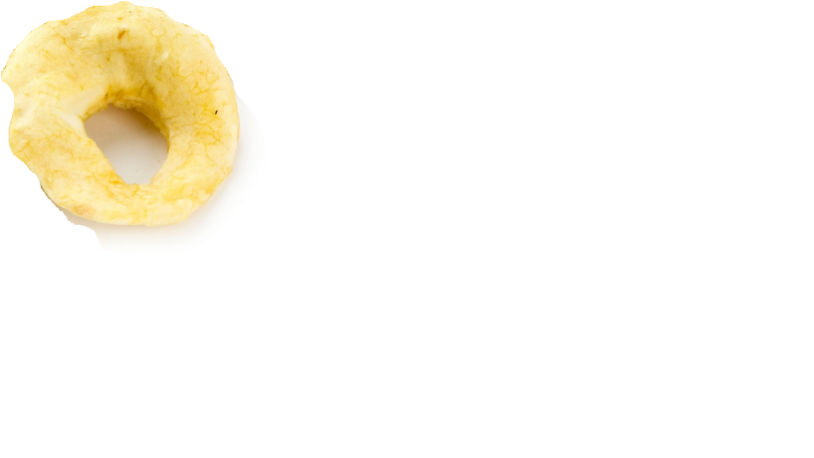 |
| --- | --- |

What would you prefer to eat?

QUESTION 8

| Dried Apricot  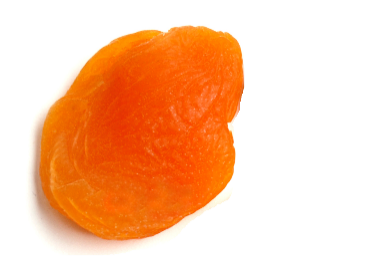 | Wholegrain cracker  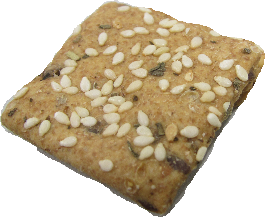 |
| --- | --- |

What would you prefer to eat?
